# Ability of dietary factors to affect homocysteine levels in mice: a review

**DOI:** 10.1186/s12986-021-00594-9

**Published:** 2021-06-30

**Authors:** Christine Brütting, Pia Hildebrand, Corinna Brandsch, Gabriele I. Stangl

**Affiliations:** Institute of Agricultural and Nutritional Sciences, Martin Luther University Halle-Wittenberg, Von-Danckelmann-Platz 2, 06120 Halle (Saale), Germany

**Keywords:** Age, Amino acids, Diet composition, Homocysteine, Mice, Sex, Strain, B vitamins

## Abstract

Homocysteine is associated with several diseases, and a series of dietary factors are known to modulate homocysteine levels. As mice are often used as model organisms to study the effects of dietary hyperhomocysteinemia, we collected data about concentrations of vitamin B_12_, vitamin B_6_, folate, methionine, cystine, and choline in mouse diets and the associated plasma/serum homocysteine levels. In addition, we more closely examined the composition of the control diet, the impact of the mouse strain, sex and age, and the duration of the dietary intervention on homocysteine levels. In total, 113 out of 1103 reviewed articles met the inclusion criteria. In the experimental and control diets, homocysteine levels varied from 0.1 to 280 µmol/l. We found negative correlations between dietary vitamin B_12_ (rho = − 0.125; *p* < 0.05), vitamin B_6_ (rho = − 0.191; *p* < 0.01) and folate (rho = − 0.395; *p* < 0.001) and circulating levels of homocysteine. In contrast, a positive correlation was observed between dietary methionine and homocysteine (methionine: rho = 0.146; *p* < 0.05). No significant correlations were found for cystine or choline and homocysteine levels. In addition, there was no correlation between the duration of the experimental diets and homocysteine levels. More importantly, the data showed that homocysteine levels varied widely in mice fed control diets as well. When comparing control diets with similar nutrient concentrations (AIN-based), there were significant differences in homocysteine levels caused by the strain (ANOVA, *p* < 0.05) and age of the mice at baseline (*r* = 0.47; *p* < 0.05). When comparing homocysteine levels and sex, female mice tended to have higher homocysteine levels than male mice (9.3 ± 5.9 µmol/l vs. 5.8 ± 4.5 µmol/l; *p* = 0.069). To conclude, diets low in vitamin B_12_, vitamin B_6_, or folate and rich in methionine are similarly effective in increasing homocysteine levels. AIN recommendations for control diets are adequate with respect to the amounts of homocysteine-modulating dietary parameters. In addition, the mouse strain and the age of mice can affect the homocysteine level.

**Supplementary Information:**

The online version contains supplementary material available at 10.1186/s12986-021-00594-9.

## Introduction

Homocysteine is a sulfur-containing essential amino acid. Its accumulation is associated with several diseases, including cardiovascular diseases such as stroke, cancer, Alzheimer’s disease and Parkinson’s disease [1]. Homocysteine is a component of one-carbon metabolism that is involved in the provision of methyl groups for biological methylation reactions. The enzyme S-adenosylmethionine synthetase catalyzes the synthesis of S-adenosylmethionine (SAM) through the reaction of methionine and adenosine triphosphate. SAM, an important methyl donor for methylation reactions, is converted to S-adenosylhomocysteine (SAH) after dispensing the methyl group. The formation of homocysteine from SAH is catalyzed by adenosylhomocysteinase. Homocysteine can be converted to methionine through the vitamin B_12_-dependent enzyme methionine synthase [2]. The acquired methyl group for remethylation comes from 5-methyltetrahydrofolate or from betaine [3]. Folate is the precursor of tetrahydrofolate [4], which is converted through methyl-tetrahydrofolate reductase to 5-methyl-tetrahydrofolate. Betaine can be formed from its precursor choline [5]. Homocysteine can also be converted to cystathionine via transsulfuration through the vitamin B_6_-dependent enzyme cystathionine β-synthase [6].

**Fig. 1:**
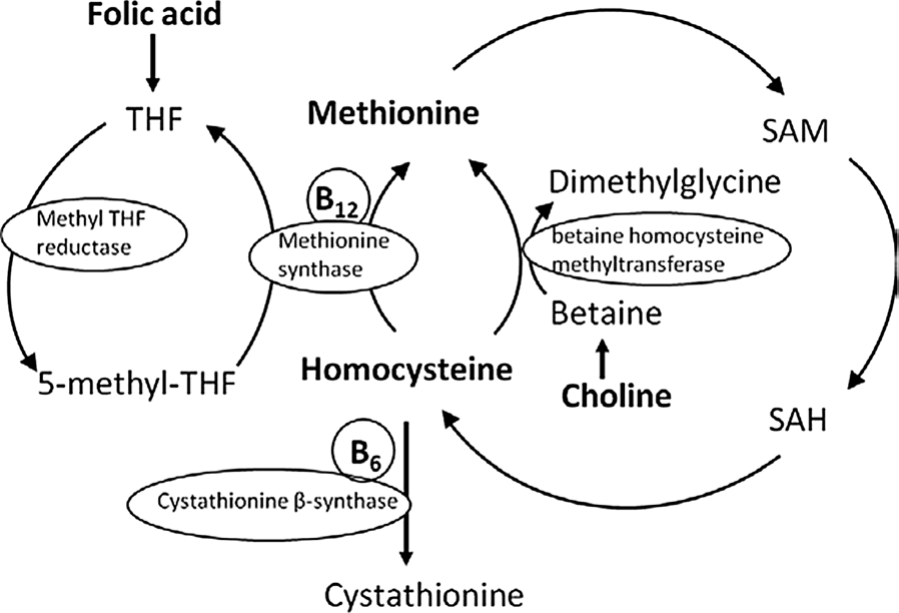
The biochemical pathways of homocysteine involving vitamin B_12_, vitamin B_6_, folate, methionine and choline. SAH—S-adenosylhomocysteine, SAM—S-adenosylmethionine, THF—tetrahydrofolate

The metabolic steps clearly show that several nutrients are involved in the one-carbon pathway and therefore can modulate homocysteine levels: methionine, vitamin B_12_, B_6_, folate and choline (Fig. 1). Thus, any excess in methionine intake or deficiencies in vitamin B_12_, B_6_, folate and choline can contribute to an increase in homocysteine levels [7].

Mice are often used as models of induced hyperhomocysteinemia and to study the impact of homocysteine on disease development. Thus, the current review evaluates different diets regarding their efficacy in increasing homocysteine levels in mice. We particularly focused on vitamin B_12_, vitamin B_6_, folate, the sulfur-containing amino acids methionine and cystine, and choline. In addition to the experimental diets, special focus was also placed on the control diets, which were used as reference. Additionally, we reviewed the impact of mouse strains, sex, age and feeding period on plasma/serum homocysteine levels. This review may be used as a reference for planning future nutrition studies on this topic.

## Methods

A systematic literature search was conducted using the database PubMed and the search items (vitamin B_12_ OR cobalamin OR vitamin B_6_ OR pyridoxine OR B vitamins OR folic acid OR folate OR folates OR homocysteine OR hyperhomocysteinemia) AND (mice OR mouse OR murine) in the title of publications. Studies were included if they met the following criteria: (I) the study was written in English and published through July 2020, (II) wild-type mice were used as the model organism, and (III) plasma or serum homocysteine levels were measured. Studies were excluded when nutrients were administered via injections, gavage or drinking water or when any kind of surgery was performed. A total of 113 studies with 305 data sets (Additional file 1: Table S1) were eligible to be included in the evaluation of this review.

The following data were extracted from each study: mouse strain, sex, age and/or body weight at baseline, duration of feeding, dietary concentrations of vitamin B_12_, vitamin B_6_, folate, the added S-containing amino acids methionine and cystine, choline and plasma or serum homocysteine levels (in the following term "plasma" is used for plasma and serum concentrations). If diet composition was not shown in the publications but was based on commercial diets, we added the manufacturer's information on nutrient contents. If diets were termed AIN-based, we used data on the composition of the AIN-93/G and AIN-93/M diets [8]. Otherwise, corresponding authors were asked for further information (which also included information regarding strain, sex or age of the mice as well as duration of dietary intervention). Correlations between plasma homocysteine levels (means and medians) and dietary compounds, age of the mice and duration of dietary intervention were analyzed using Pearson’s correlation testing since variables are normally distributed and Spearman correlation since variables are not normally distributed. Differences between plasma homocysteine levels and sex variables were analyzed using Student’s t test, and strain differences were analyzed with Levene’s test to assess homogeneity of variances and single-factor analysis of variance (ANOVA) followed by Hochberg’s GT2 post hoc test (SPSS 2020).

## Results

### Dietary parameters

**Fig. 2:**
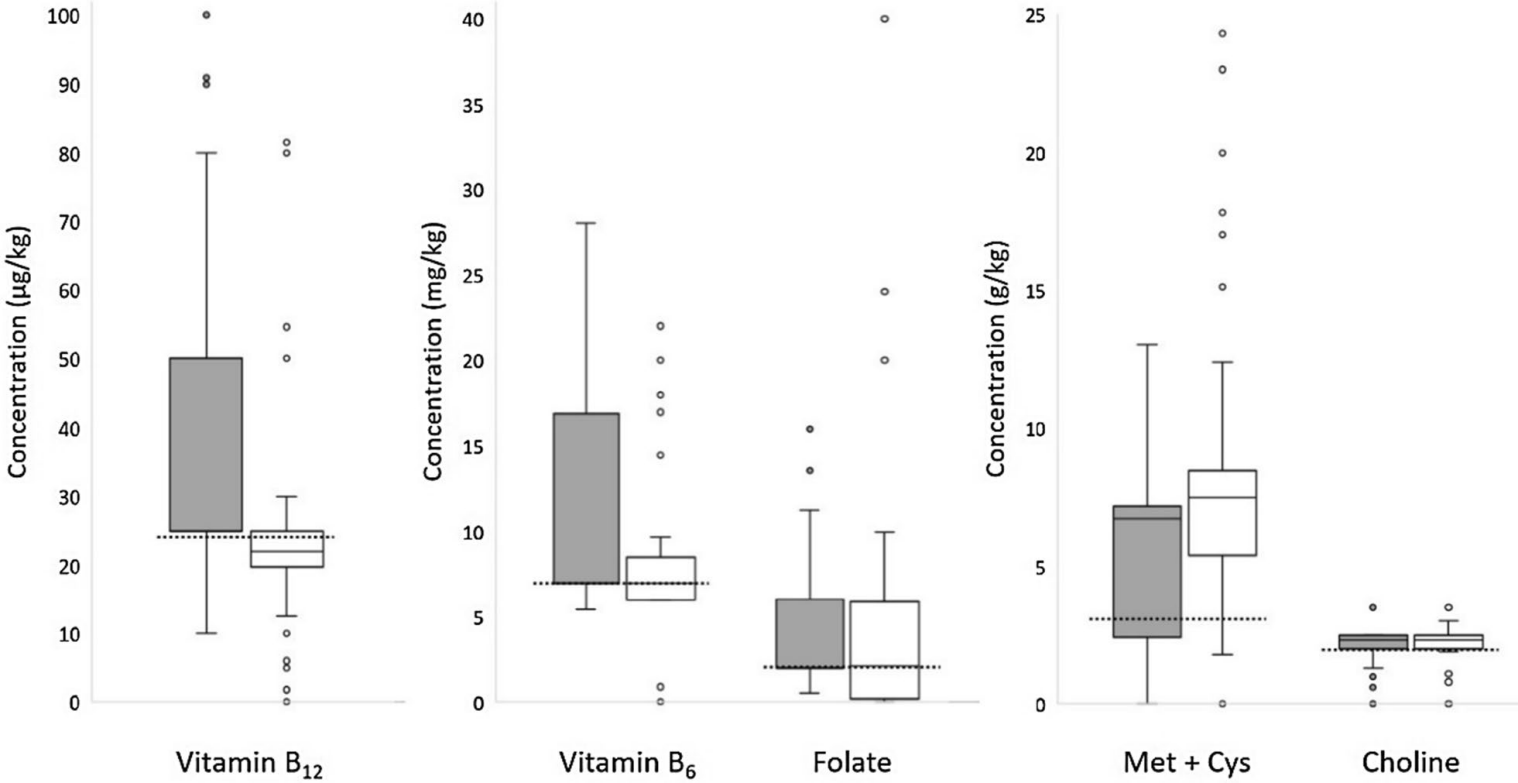
Boxplots show medians, interquartile ranges and 1.5 × interquartile ranges of concentrations of vitamin B_12_ (µg/kg), vitamin B_6_ (mg/kg), folate (mg/kg), methionine + cystine (g/kg) and choline (g/kg) in control (gray box, n = 118 data sets out of 85 studies) and experimental (white box, *n* = 137 data sets out of 56 studies) diets, which were used to increase plasma levels of homocysteine. AIN-93-based nutrient recommendations [8] for control diets are depicted as dashed lines for each nutrient. For vitamin B_12_, vitamin B_6_ and folate the medians coincide with the 25th percentile

In 56 out of 113 studies, the composition of the experimental diets was described in detail. Experimental diets had vitamin B_12_ concentrations varying from 0 to 81.6 µg/kg diet, vitamin B_6_ concentrations varying from 0 to 22 mg/kg diet, folate concentrations varying from 0 to 40 mg/kg diet, methionine + cystine concentrations varying from 0 to 24.3 g/kg diet, and choline concentrations varying from 0 to 3.5 g/kg diet (Fig. 2).

In addition to the experimental diets, we also evaluated the control diets that were used as a reference, especially with regard to their potential to minimize homocysteine levels. In 85 out of 113 studies, the composition of the control diets was described in detail. Studies have shown high variations in homocysteine-relevant nutrients in control diets. The concentration of vitamin B_12_ varied from 10 to 100 µg/kg diet, that of vitamin B_6_ from 5.4 to 28 mg/kg diet, that of folate from 0.5 to 16 mg/kg diet, that of methionine and cysteine from 0 to 13 g/kg diet, and that of choline from 0 to 3.5 g/kg diet (Fig. 2). Compared to nutrient recommendations for mice [8], concentrations of vitamin B_12_, vitamin B_6_, folate and S-containing amino acids in the control diets used were often markedly higher (Fig. 2). However, AIN-based control diets were administered in only 14 out of 113 studies (Table 1 and Additional file 1: Table S1).

**Table 1:** Plasma homocysteine levels in mice fed AIN-93-based control diets

Mouse strain	Sex	Age at baseline	Duration (weeks)	Plasma Hcy (µmol/l)	*n*	References
129/Sv	f + m	3 wks	6	0.1^#^	15	[9]
129/Sv	f + m	3 wks	9	0.1^#^	15	[9]
129/Sv	f + m	3 wks	9–13	2.0 ± 0.6^b^	nda	[10]
CD-1	f + m	Adult	9	2.5^#^	13	[11]
C57BL/6	m	6 wks	8	2.5 ± 0.7^b^	nda	[12]
C57BL/6	m	6 wks	4	2.6 ± 0.8^b^	nda	[12]
129/Sv	f + m	3 wks	9	2.8 ± 0.3^nda^	nda	[13]
Swiss	m	3 wks	27	3.0 ± 0.4^b^	6–8	[14]
C57BL/6	m	3 wks	5	3.0 ± 2.2^c^	nda	[15]
C57BL/6	m	6 wks	8	3.3 ± 0.8^b^	6	[16]
C57BL/6	f	8 wks	9	3.6 ± 0.7^b^	15	[17]
SAMP8	m	13 wks	4	4.0^#^	nda	[18]
BALB/c	f	17 wks	2	5.2 ± 0.2^b^	23	[19]
Swiss	m	3 wks	10	5.2 ± 0.6^b^	6–8	[14]
C57BL/6	f	3 wks	5	5.4 ± 1.7^c^	nda	[15]
C57BL/6	f	6–8 wks	7	5.5 ± 5.4^c^	10	[15]
SAMP8	m	17 wks	26	6.5^#^	15	[18]
SAMR1	m	17 wks	26	6.5^#^	nda	[18]
Swiss	f	3 wks	10	7.1 ± 0.7^b^	6–8	[14]
Swiss	f	3 wks	27	8.1 ± 0.8^b^	6–8	[14]
Swiss	m	3 wks	1	8.7 ± 0.9^b^	6–8	[14]
Swiss	f	Adult	3	9.2^#^	6–8	[14]
Swiss	f	3 wks	1	9.7 ± 0.6^b^	6–8	[14]
C57BL/6	f	7 wks	2	16.7 ± 1.5^a^	20	[20]
BALB/c	m	17 wks	52	18^#^	nda	[19]
BALB/c	f	17 wks	52	22.5^#^	nda	[19]

f, female; m, male; *n*, number of included mice; nda, no data available; wks, weeks

^a^Mean ± standard deviation

^b^Mean ± standard error

^c^Median ± interquartile range

^#^Read from diagrams of data from mice that received AIN-93-based control diets containing 25 µg vitamin B_12_, 7 mg vitamin B_6_, 2 mg folate, 3 g methionine + cystine and 2.5 g choline per kg diet; only studies with complete data sets about mouse strain, sex, age at baseline, and the duration of feeding (in weeks) were included

**Fig. 3:**
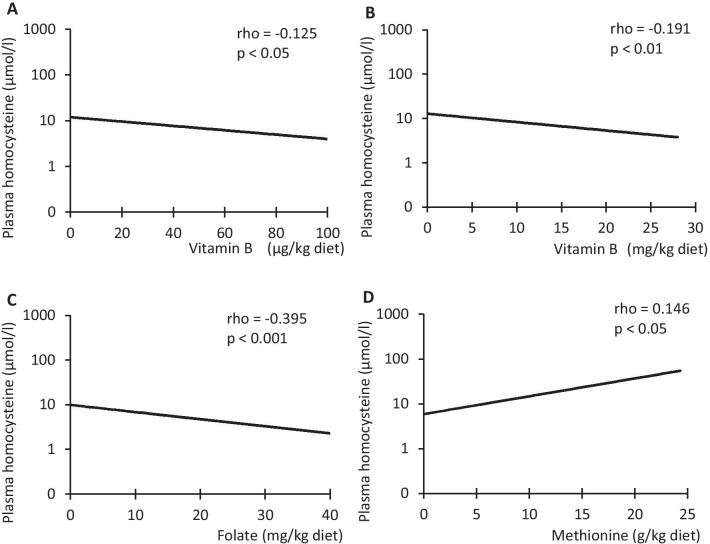
Correlations between the diet ingredients vitamin B_12_ (**A**), vitamin B_6_ (**B**), folate (**C**) as well as methionine (**D**) and the plasma homocysteine level (logarithmic scale) of mice fed experimental or control diets; Spearman correlation (rho) was performed, since variables are not normally distributed; *n* = 255 data sets

In mice fed the experimental or control diets, circulating homocysteine levels varied from 0.1 to 280 µmol/l. Analysis of the associations between components of the experimental and control diets and plasma homocysteine levels revealed negative correlations for vitamin B_12_ (rho = − 0.125; *p* < 0.05), vitamin B_6_ (rho = − 0.191; *p* < 0.01) and folate (rho = − 0.395; *p* < 0.001; Fig. 3). A positive correlation was observed between dietary methionine and plasma homocysteine levels (methionine: rho = 0.146; *p* < 0.05; Fig. 3). No significant correlations were found for homocysteine levels and dietary cystine (rho = − 0.076; *p* > 0.05) or choline (rho = 0.044; *p* > 0.05). The duration of the analyzed feeding experiments varied between 3 and 17 weeks. However, there was no correlation between feeding duration and plasma homocysteine level (*r* = − 0.05; *p* > 0.5).

### Strain, sex, and age of mice

When comparing the circulating homocysteine levels in mice resulting from all analyzed control diets (including AIN-based control diets), we found varying homocysteine levels ranging from 0.1 to 24.1 µmol/l (Additional file 1: Table S1). Surprisingly, homocysteine levels in mice consuming strictly AIN-based control diets also varied in a wide range (from 0.1 to 22.5 µmol/l; Table 1), indicating that parameters other than nutrients influenced homocysteine levels.

**Fig. 4:**
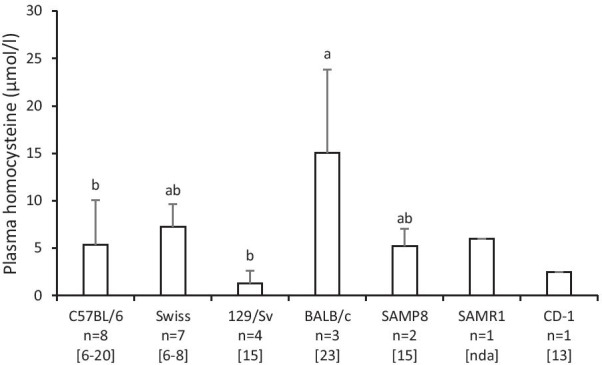
Circulating plasma homocysteine levels (µmol/l) in mice of different strains that received AIN-93-based control diets containing 25 µg vitamin B_12_, 7 mg vitamin B_6_, 2 mg folate, 3 g methionine + cystine and 2.5 g choline per kg diet; mean + standard deviation, when more than one data set was available; different letters indicate a statistically significant difference (*p* < 0.05, ANOVA followed by Hochberg’s GT2 post hoc test); n, number of included data sets (Table 1); number of integrated animals per study (ranges) are stated in square brackets; nda, no data available

In mice that received AIN-93-based control diets (Table 1), there were differences in homocysteine levels related to the strain (*p* < 0.05; Fig. 4) and age of the mice at baseline (r = 0.474; *p* < 0.05). When comparing homocysteine levels and sex, female mice tended to have higher homocysteine levels than male mice (9.3 ± 5.9 µmol/l vs. 5.8 ± 4.5 µmol/l; *p* = 0.069, Table 1).

## Discussion

The current review shows that hyperhomocysteinemia can be induced by numerous different dietary interventions, such as a reduction in vitamin B_6_, vitamin B_12_ or folate concentration and an increase in methionine concentration. Study data showed that dietary cystine and choline had no effects on plasma homocysteine levels in mice. In addition, there was no correlation between the duration of feeding the experimental diets and plasma homocysteine levels. When diets were fed over varying periods, in most cases, there was no difference between homocysteine levels at different time points (Additional file 1: Table S1). One study found differences after 2 weeks of feeding the experimental diets, but no differences between 2 and up to 10 weeks. Thus, dietary interventions to increase homocysteine levels appear to be rapidly effective.

The type of control diet used in these studies showed great variations (Additional file 1: Table S1). The intake of AIN-93-based diets resulted in homocysteine levels similar to those of the other control diets. Hence, higher doses of vitamin B_12_, vitamin B_6_ and folate than recommended in the AIN-93 diet [8] do not seem to further decrease homocysteine levels. However, it should be mentioned that AIN-based control diets were only used in 14 out of 113 studies (Additional file 1: Table S1).

Plasma levels of homocysteine depend on the mouse strain because the growth rate of mice and thus the nutrient requirements depend on the genetic background [21]. Older mice have higher homocysteine levels than younger mice, which is in line with homocysteine data in humans [22, 23]. An age-related reduction in renal function is attributable to this effect [24]. In addition, females tend to have higher homocysteine levels than males. It is assumed that the renal activity of cystathionine β-synthase, which catalyzes an important step in the formation of cysteine from homocysteine, is regulated by testosterone [25] and thus is commonly higher in males than in females [26].

In addition, it must be kept in mind that the different methods used for quantification of homocysteine such as chromatography, immunoassays or capillary electrophoresis could have influenced the results [27]. In our review, the high-performance liquid chromatography (HPLC) was the most frequently used method to quantify plasma homocysteine (in 72 out of 113 studies, Additional file 1: Table S1).

To conclude, vitamin B_12_, vitamin B_6_, folate, and methionine are similarly effective in reducing homocysteine levels. AIN recommendations for control diets are adequate with respect to the amounts of homocysteine-modulating dietary parameters. In addition to dietary parameters, the mouse strain and the age of mice can affect homocysteine levels.

## Supplementary Information

**Additional file 1**. **Supplemental Table:** Diet composition and mouse data.

## Data Availability

All data analysed during this study are included in this published article and its supplementary information file.

## Abbreviations

ANOVA: Analysis of variance
SAH: S-adenosylhomocysteine
SAM: S-adenosylmethionine
