# Supplementary material for: Ability of dietary factors to affect homocysteine levels in mice: a review
Source: Nutr Metab (Lond). 2021 Jun 30;18:68. doi: 10.1186/s12986-021-00594-9 (PMC8243555; doi:10.1186/s12986-021-00594-9)
Supplement: Supplementary file 1 — Additional file 1. Supplemental Table: Diet composition and mouse data. [file 12986_2021_594_MOESM1_ESM.docx]

**Supplemental Table: Diet composition and mouse data**

| **Mouse** | **Sex** | **Age / weight** | **Duration** | **Diet** | **B_12_** | **B_6_** | **Folate** | **Methionine** | **Cystine** | **Choline** | **Hcy** | **Detection** | **Reference** |
| --- | --- | --- | --- | --- | --- | --- | --- | --- | --- | --- | --- | --- | --- |
| **strain** |  | **at baseline** | **(wks)** |  | **(µg/kg)** | **(mg/kg)** | **(mg/kg)** | **(g/kg)** | **(g/kg)** | **(g/kg)** | **(µmol/l)** | **method** |  |
| **Nutrient Requirements of the Mouse** | | | |  |  |  |  |  |  |  |  |  |  |
|  |  |  | **AIN-93G** | | 25 | 7 | 2 | 0 | 3 | 2.5 |  |  | [8] |
|  |  |  | **AIN-93M** | | 25 | 7 | 2 | 0 | 1.8 | 2.5 |  |  | [8] |
| **Studies including one mouse strain** | | |  |  |  |  |  |  |  |  |  |  |  |
| BALB/c | f | weanling | 8 | c | 25 | 7 | 2 | 3.3 | 3.5 | 2.5 | 20^#^ | IA | [28] |
| BALB/c | f | weanling | 8 | e | 25 | 7 | 0.3 | 3.3 | 3.5 | 2.5 | 60^#^ | IA |  |
| BALB/c | f | weanling | 8 | c | 25 | 7 | 2 | 3.3 | 3.5 | 2.5 | 14^#^ | HPLC | [29] |
| BALB/c | f | weanling | 8 | e | 25 | 7 | 40 | 3.3 | 3.5 | 2.5 | 17^#^ | HPLC |  |
| BALB/c | f | weanling | 5 | c | 50 | 6 | 5.9 | 4.3 | 3.1 | 2.3 | 3.3 ± 0.8 | HPLC | [30] |
| BALB/c | m | weanling | 5 | c | 50 | 6 | 5.9 | 4.3 | 3.1 | 2.3 | 3.2 ± 1.2 | HPLC |  |
| BALB/c | nda | weanling | 7-15 | c | 91 | 16.9 | 6.7 | 4 | 3 | 1.7 | 3^#^ | HPLC | [31] |
| BALB/c | nda | weanling | 7-15 | e1 | 25 | 8.5 | 0.2 | 4.1 | 3 | 0.8 | 8.4 ± 3.1 | HPLC |  |
| BALB/c | nda | weanling | 7-15 | e2 | 25 | 8.5 | 0.2 | 8.2 | 3 | 0.8 | 9.5 ± 1.7 | HPLC |  |
| BALB/c | nda | adult | 13 | c | 90 | 17 | 7 | 4 | 3 | 2.2 | 2.9 ± 0.4 | IA | [32] |
| BALB/c | m | 5 wks | 4 | c | 20 | 7 | 2 | 0 | 1.8 | 2.5 | 3.53 ± 0.81 | HPLC | [33] |
| BALB/c | f | 17 wks | 2 | c | 25 | 7 | 2 | 0 | 1.8 | 2.5 | 5.2 ± 0.2 | HPLC | [19] |
| BALB/c | f | 17 wks | 52 | c | 25 | 7 | 2 | 0 | 1.8 | 2.5 | 22.5^#^ | HPLC |  |
| BALB/c | m | 17 wks | 52 | c | 25 | 7 | 2 | 0 | 1.8 | 2.5 | 18^#^ | HPLC |  |
| BALB/c | f | weanling | 6 | c | 25 | 7 | 2 | 3.3 | 3.5 | 2.5 | 13^#^ | HPLC | [34] |
| BALB/c | f | weanling | 6 | e | 25 | 7 | 20 | 3.3 | 3.5 | 2.5 | 17^#^ | HPLC |  |
| BALB/c | f | weanling | 48-52 | c | 25 | 7 | 2 | 3.3 | 3.5 | 2.5 | 15.2 ± 1.4 | IA | [35] |
| BALB/c | m | weanling | 48-52 | c | 25 | 7 | 2 | 3.3 | 3.5 | 2.5 | 24.1 ± 5.6 | IA |  |
| BALB/c | f | weanling | 48-52 | e | 25 | 7 | 0.3 | 3.3 | 3.5 | 2.5 | 51.1 ± 11.3 | IA |  |
| BALB/c | m | weanling | 48-52 | e | 25 | 7 | 0.3 | 3.3 | 3.5 | 2.5 | 57.2 ± 13.5 | IA |  |
| BALB/c | f | 17 wks | 8 | c | 25 | 7 | 2 | 3.3 | 3.5 | 2.5 | 13.8 ± 1.6 | HPLC | [36] |
| BALB/c | f | 17 wks | 8 | e | 25 | 7 | 0.3 | 3.3 | 3.5 | 2.5 | 25^#^ | HPLC |  |
| BALB/cAnN | f | 17 wks | 6-10 | c | 25 | 7 | 2 | 3.3 | 3.5 | 2.5 | 12.3 ± 1.1 | HPLC | [37] |
| BALB/cAnN | f | 17 wks | 6-10 | e | 25 | 7 | 10 | 3.3 | 3.5 | 2.5 | 11.5 ± 1.2 | HPLC |  |
| BALB/cAnN | f | 17 wks | 15-19 | c | 25 | 7 | 2 | 3.3 | 3.5 | 2.5 | 12^#^ | HPLC | [38] |
| BALB/cAnN | f | 17 wks | 15-19 | e | 25 | 7 | 10 | 3.3 | 3.5 | 2.5 | 18^#^ | HPLC |  |
| B6SJL | nda | 6 wks | 11 | c | nda | nda | nda | nda | nda | nda | 3.8 ± 0.26 | HPLC | [39] |
| CBA | m | 8 wks | 4 | c | nda | nda | 8 | nda | nda | nda | 5.4 ± 0.5 | HPLC | [40] |
| CBA | m | 8 wks | 4 | e | nda | nda | 0 | nda | nda | nda | 12.7 ± 1.2 | HPLC |  |
| CD-1 | f + m | adult | 9 | c | 25 | 7 | 2 | 0 | 1.8 | 2.5 | 2.5^#^ | GC | [11] |
| CD-1 | f + m | adult | 9 | e | 0 | 7 | 0 | 0 | 1.8 | 2.5 | 14.8^#^ | GC |  |
| CD-1 | m | weanling | 9 | c | 25 | 7 | 2 | 8.2 | 3.5 | 2.5 | 1.3 ± 0.1 | HPLC | [41] |
| CD-1 | f + m | 30–40 g | 7 | c | 24 | 9 | 2 | 2.7 | 3.2 | 0.6 | 2.8 ± 1.5 | GC | [42] |
| C57 | f | 14 wks | 11 | c | 25 | 7 | 2 | 3 | 0 | 2 | 8^#^ | HPLC | [43] |
| C57 | f | 14 wks | 11 | e | 25 | 7 | 0 | 3 | 0 | 2 | 17^#^ | HPLC |  |
| C57BL/KsJ | f | 26 wks | 1 | c | 33 | 17 | 7.9 | 4.9 | 3.5 | 2.2 | 7.1 ± 1.3 | HPLC | [44] |
| C57BL/KsJ | f | 26 wks | 2 | c | 33 | 17 | 7.9 | 4.9 | 3.5 | 2.2 | 7.2 ± 1.1 | HPLC |  |
| C57BL/KsJ | f | 26 wks | 3 | c | 33 | 17 | 7.9 | 4.9 | 3.5 | 2.2 | 6.9 ± 1.2 | HPLC |  |
| C57BL/KsJ | f | 26 wks | 4 | c | 33 | 17 | 7.9 | 4.9 | 3.5 | 2.2 | 7.0 ± 1.0 | HPLC |  |
| C57BL/6 | f | 3-4 wks | 4 | c | 16 | 7.1 | 2.1 | 3 | 0.6 | 2 | 3.9 ± 0.56 | HPLC | [45] |
| C57BL/6 | f | 3-4 wks | 4 | e | 6 | 7.1 | 2.1 | 3 | 0.6 | 2 | 8 ± 0.57 | HPLC |  |
| C57BL/6 | f | 3-4 wks | 12 | c | 16 | 7.1 | 2.1 | 3 | 0.6 | 2 | 4.2 ± 0.4 | HPLC |  |
| C57BL/6 | f | 3-4 wks | 12 | e | 6 | 7.1 | 2.1 | 3 | 0.6 | 2 | 8.7 ± 0.78 | HPLC |  |
| C57BL/6 | m | 8 wks | 12 | c | 80 | 18 | 4 | 4 | 3 | 1.2 | 3.3^#^ | HPLC | [46] |
| C57BL/6 | m | 8 wks | 12 | e | 29.7 | 22 | 2 | 3 | 0 | 3 | 5.3^#^ | HPLC |  |
| C57BL/6J | m | 25 g | 3 | c | 25 | 7 | 2 | 5.2 | 3 | 1 | 4.8 ± 0.2 | HPLC | [47] |
| C57BL/6J | m | 25 g | 3 | e1 | 25 | 7 | 2 | 5.2 | 3 | 3.5 | 4.4 ± 0.4 | HPLC |  |
| C57BL/6J | m | 25 g | 3 | e2 | 25 | 7 | 2 | 5.2 | 3 | 0 | 4.2 ± 0.2 | HPLC |  |
| C57BL6/J | m | 3-4 wks | 10 | c | 25 | 7 | 2 | 3.3 | 1.8 | 2.5 | 5.2 ± 0.7 | HPLC | [48] |
| C57BL/6J | m | 3-4 wks | 10 | e1 | 0 | 0 | 0 | 3.3 | 1.8 | 2.5 | 35.2 ± 28.7 | HPLC |  |
| C57BL/6J | m | 3-4 wks | 10 | e2 | 25 | 7 | 2 | 13.3 | 1.8 | 2.5 | 13.9 ± 3.6 | HPLC |  |
| C57BL/6J | nda | 9 wks | 6 | c | 10 | 9.7 | 0.5 | 5 | 2 | 2 | 3 ± 1.5 | HPLC | [49] |
| C57BL/6J | nda | 9 wks | 6 | e | 10 | 9.7 | 0.5 | 15 | 2 | 2 | 51.8 ± 22.7 | HPLC |  |
| C57BL/6 | m | 6 wks | 6-7 | c | 90 | 17 | 7 | 4 | 3 | 2.2 | 6.6 ± 1.9 | HPLC | [50] |
| C57BL/6 | m | 6 wks | 6-7 | e | 0 | 0 | 0 | 7.7 | 0 | 2.5 | 89.2 ± 48.9 | HPLC |  |
| C57BL/6 | f | 8 wks | 12 | c | nda | nda | nda | nda | nda | nda | 13^#^ | IA | [51] |
| C57BL/6 | f | 8 wks | 12 | e | nda | nda | nda | nda | nda | nda | 31^#^ | IA |  |
| C57BL/6J | nda | 3-4 wks | 6 | c | 90 | 17 | 7.5 | 4 | 3 | 2.2 | 5.3 ± 0.7 | HPLC | [52] |
| C57BL/6J | nda | 3-4 wks | 6 | e | nda | nda | 1.5 | nda | nda | nda | 11.6 ± 4.5 | HPLC |  |
| C57BL/6J | f + m | 3-4 wks | 25-34 | c | 90 | 17 | 6.7 | 4 | 3 | 2.2 | 3.6 ± 0.2 | HPLC | [53] |
| C57BL/6J | f + m | 3-4 wks | 25-34 | e | 25 | 8.5 | 0.2 | 8.2 | 0 | 2.0 | 14.4 ± 1.1 | HPLC |  |
| C57BL6/J | f + m | 3-4 wks | 16-17 | c | 90 | 17 | 7 | 4 | 3 | 2.2 | 4.3 ± 0.6 | AAA | [54] |
| C57BL6/J | m | 4 wks | 21-47 | c | 90 | 17 | 6.7 | 4 | 3 | 2.2 | 4.5^#^ | HPLC | [55] |
| C57BL/6J | m | 4 wks | 21-47 | e | 25 | 8.5 | 0.2 | 8.2 | 0 | 2 | 10.5^#^ | HPLC |  |
| C57BL/6J | f + m | 8 wks | 8 | c | 54.6 | 14.5 | 3.2 | 3.7 | 3 | 2.2 | 3.7 ± 1.2 | HPLC | [56] |
| C57BL/6J | f + m | 8 wks | 8 | e | 54.6 | 14.5 | 3.2 | 20 | 3 | 2.2 | 40.7 ± 18.9 | HPLC |  |
| C57BL/6J | f + m | 3 wks | 7 | c | 90 | 17 | 6.7 | 4 | 3 | 2.2 | 5.7 ± 2.1 | GC | [57] |
| C57BL/6J | f + m | 3 wks | 7 | e | 25 | 8.5 | 0.2 | 8.2 | 0 | 2 | 21.4 ± 3.9 | GC |  |
| C57BL/6J | nda | nda | 8 | c | nda | nda | nda | nda | nda | nda | 4.1 ± 0.2 | IA + HPLC | [58] |
| C57BL/6J | nda | nda | 8 | e | 10.4 | 0.01 | 0.1 | 7.7 | 0 | 2.5 | 23.5 ± 5 | IA + HPLC |  |
| C57BL/6J | nda | 3-4 wks | 3-12 | c | 25 | 8.5 | 4.2 | 0 | 3 | 0.8 | 2.5 ± 0.8 | HPLC | [59] |
| C57BL/6J | nda | 3-4 wks | 3-12 | e1 | 25 | 8.5 | 0.2 | 0 | 3 | 0.8 | 7 ± 1.1 | HPLC |  |
| C57BL/6J | nda | 3-4 wks | 3-12 | e2 | 25 | 8.5 | 4.2 | 4.1 | 3 | 0.8 | 6.2 ± 0.9 | HPLC |  |
| C57BL/6J | m | 6 wks | 10 | c | nda | nda | nda | nda | nda | nda | 6^#^ | IA | [60] |
| C57BL/6J | m | 6 wks | 10 | e | nda | nda | nda | nda | nda | nda | 12^#^ | IA |  |
| C57BL/6J | m | adult, 20-25 g | 4 | c | nda | nda | nda | nda | nda | nda | 5 ± 2 | HPLC | [61] |
| C57BL/6J | m | 3 wks | 11 | c | 80 | 18 | 4 | 5 | 3 | 1.2 | 9.5^#^ | IA | [62] |
| C57BL/6J | m | 3 wks | 11 | e | 80 | 18 | 24 | 5 | 3 | 1.2 | 6.5^#^ | IA |  |
| C57BL/6 | f + m | 6 wks | 4 | c | 10.1 | 7.1 | 2 | 3 | 0.6 | 1.1 | 4.6 ± 0.4 | HPLC | [63] |
| C57BL/6 | f + m | 6 wks | 4 | e1 | 10.1 | 7.1 | 0 | 3 | 0.6 | 1.1 | 6^#^ | HPLC |  |
| C57BL/6 | f + m | 6 wks | 4 | e2 | 10.1 | 7.1 | 20 | 3 | 0.6 | 1.1 | 4^#^ | HPLC |  |
| C57BL6/J | f | nda | 24 | c | 50 | 20 | 3 | 3 | 3.4 | 2.5 | 4.8^#^ | GC | [64] |
| C57BL/6J | f | nda | 24 | e | 0 | 20 | 3 | 3 | 3.4 | 2.5 | 23^#^ | GC |  |
| C57BL/6J | m | nda | 24 | c | 50 | 20 | 3 | 3 | 3.4 | 2.5 | 5^#^ | GC |  |
| C57BL/6J | m | nda | 24 | e | 0 | 20 | 3 | 3 | 3.4 | 2.5 | 30^#^ | GC |  |
| C57BL/6J | f | 4 wks | 2 | c | nda | nda | nda | nda | nda | nda | 3.8 ± 0.8 | HPLC | [65] |
| C57BL/6J | f | 4 wks | 2 | e1 | nda | nda | nda | nda | nda | nda | 6 ± 1.8 | HPLC |  |
| C57BL/6J | f | 4 wks | 2 | e2 | nda | nda | nda | nda | nda | nda | 2.4 ± 0.6 | HPLC |  |
| C57BL/6J | f | 4 wks | 10 | c | nda | nda | nda | nda | nda | nda | 2.9 ± 0.8 | HPLC |  |
| C57BL/6J | f | 4 wks | 10 | e1 | nda | nda | nda | nda | nda | nda | 3.6 ± 1.2 | HPLC |  |
| C57BL/6J | f | 4 wks | 10 | e2 | nda | nda | nda | nda | nda | nda | 1.5 ± 0.7 | HPLC |  |
| C57BL/6J | f | 4 wks | 20 | c | nda | nda | nda | nda | nda | nda | 2.5 ± 0.6 | HPLC |  |
| C57BL/6J | f | 4 wks | 20 | e1 | nda | nda | nda | nda | nda | nda | 2.7 ± 1 | HPLC |  |
| C57BL/6J | f | 4 wks | 20 | e2 | nda | nda | nda | nda | nda | nda | 1.6 ± 0.5 | HPLC |  |
| C57BL/6 | nda | nda | nda | c | nda | nda | nda | nda | nda | nda | 3.4 ± 0.3 | HPLC | [66] |
| C57BL/6 | f | 6-8 wks | 7 | c | 25 | 7 | 2 | 0 | 1.8 | 2.5 | 5.5 ± 5.4 | CHEM | [15] |
| C57BL/6 | f | 6-8 wks | 7 | e1 | 5 | 7 | 8 | 0 | 1.8 | 2.5 | 9.8 ± 3 | CHEM |  |
| C57BL/6 | f | 3 wks | 5 | c | 25 | 7 | 2 | 0 | 3 | 2.5 | 5.4 ± 1.7 | CHEM |  |
| C57BL/6 | f | 3 wks | 5 | e2 | 5 | 7 | 8 | 0 | 3 | 2.5 | 4.1 ± 3.1 | CHEM |  |
| C57BL/6 | m | 3 wks | 5 | c | 25 | 7 | 2 | 0 | 3 | 2.5 | 3.0 ± 2.2 | CHEM |  |
| C57BL/6 | m | 3 wks | 5 | e2 | 5 | 7 | 8 | 0 | 3 | 2.5 | 2.7 ± 1.6 | CHEM |  |
| C57BL/6 | m | 8 wks | 24 | c | 30 | 8 | 2 | 0 | 1.8 | 2.5 | 3.4 (2.1–4.0) | HPLC | [67] |
| C57BL/6 | m | 8 wks | 24 | e | 1.8 | 0.2 | 0.1 | 0 | 1.8 | 2.5 | 9.4 (5.3–12.5) | HPLC |  |
| C57BL/6J | f + m | 3 wks | 12 | c | 50 | 20 | 3 | 3 | 0 | 2.5 | 4^#^ | HPLC | [68] |
| C57BL/6J | f + m | 3 wks | 12 | e | 0 | 20 | 3 | 3 | 0 | 2.5 | 8^#^ | HPLC |  |
| C57BL/6J | f + m | 3 wks | 29 | c | 50 | 20 | 3 | 3 | 0 | 2.5 | 8^#^ | HPLC |  |
| C57BL/6J | f + m | 3 wks | 29 | e | 0 | 20 | 3 | 3 | 0 | 2.5 | 16^#^ | HPLC |  |
| C57BL/6 | m | 8 wks | 24 | c | nda | nda | nda | nda | nda | nda | 4^#^ | IA | [69] |
| C57BL/6 | m | 8 wks | 24 | e | nda | nda | nda | nda | nda | nda | 4.2^#^ | IA |  |
| C57BL/6J | m | 9 wks | 12 | c | nda | nda | nda | nda | nda | nda | 8^#^ | IA | [70] |
| C57BL/6 | m | 9 wks | 12 | e | nda | nda | nda | nda | nda | nda | 20^#^ | IA |  |
| C57BL/6J | m | 4 wks | 6 | c | nda | nda | 2 | nda | nda | nda | 11^#^ | IA | [71] |
| C57BL/6J | m | 6 wks | 16 | c | nda | nda | nda | nda | nda | nda | 5.4 ± 0.7 | IA | [72] |
| C57BL/6 | m | 20 g | nda | c | 41 | 11 | 13.6 | 3.3 | 3.7 | 3.5 | 2.5^#^ | IA | [73] |
| C57BL/6J | f + m | 4 wks | 8 | c | 50 | 17 | 6.1 | 5.6 | 4.2 | 2.2 | 7.4 ± 2.2 | HPLC | [74] |
| C57BL/6 | m | 13 wks | 2 | c | 100 | 28 | 8 | 0 | 3 | 2 | 7.2 ± 0.3 | GC | [75] |
| C57BL/6 | f | 8 wks | 9 | c | 25 | 7 | 2 | 0 | 2.5 | 2.5 | 3.6 ± 0.7 | HPLC | [17] |
| C57BL/6 | m | 6 wks | 8-10 | c | 51 | 9.6 | 3 | 4.2 | 2.8 | 2 | 11.3 ± 1 | IA | [76] |
| C57BL/6 | m | 6 wks | 8-10 | e1 | 29.7 | 22 | 2 | 4.3 | 0 | 3.5 | 17.3 ± 1.4 | IA |  |
| C57BL/6 | m | 6 wks | 8-10 | e2 | 29.7 | 22 | 2 | 24.3 | 0 | 3.5 | 69.5 ± 5.9 | IA |  |
| C57BL/6J | m | 7 wks | 10 | c | 25 | 7 | 2 | 3.6 | 1.8 | 2.5 | 6^#^ | HPLC | [77] |
| C57BL/6J | m | 7 wks | 10 | e | 25 | 7 | 0 | 1.8 | 1.8 | 0 | 38^#^ | HPLC |  |
| C57BL/6J | m | 8 wks | 10 | c | 25 | 7 | 2 | 3 | 0 | 2 | 5^#^ | IA | [78] |
| C57BL/6J | m | 8 wks | 10 | e | 25 | 7 | 1.5 | 20 | 0 | 2 | 88^#^ | IA |  |
| C57BL/6 | nda | 3.5 wks | 1.5 | c | 10.1 | 7.1 | 2.1 | 3.0 | 0.6 | 1.1 | 6.5 ± 0.7 | HPLC | [79] |
| C57BL/6J | f + m | 26-39 wks | 2 | c | 10 | 9.7 | 0.5 | 5 | 2 | 2 | 3.5 ± 0.3 | HPLC | [80] |
| C57BL/6J | f + m | 26-39 wks | 2 | e | 10 | 9.7 | 0.5 | 15 | 2 | 2 | 126 ± 76 | HPLC |  |
| C57BL/6 | m | 6 wks | 8 | c | nda | nda | 2 | 3.9 | nda | 3 | 2.3^#^ | HPLC | [81] |
| C57BL/6J | m | 6 wks | 8 | e | nda | nda | 0.4 | 3.9 | nda | 1.5 | 3^#^ | HPLC |  |
| C57BL/6J | m | 13-17 wks | 6 | c | 51 | 10 | 1.9 | 6.2 | 3.6 | 2 | 5^#^ | nda | [82] |
| C57BL/6J | m | nda | 12 | c | nda | nda | nda | nda | nda | nda | 3.0 ± 0.2 | IA | [83] |
| C57BL/6J | f | 4 wks | 8 | c | 50 | 17 | 6.1 | 6.6 | 4.2 | 2.2 | 7.4 ± 2.2 | HPLC | [84] |
| C57BL/6J | f | 4 wks | 8 | c | 50 | 17 | 6.1 | 6.6 | 4.2 | 2.2 | 7.4 ± 2.2 | HPLC | [85] |
| C57BL/6J | f | 4 wks | 8 | c | 50 | 17 | 6.1 | 6.6 | 4.2 | 2.2 | 7.4 ± 2.2 | HPLC | [86] |
| C57BL/6J | f | 4 wks | 8 | c | 50 | 17 | 6.1 | 6.6 | 4.2 | 2.2 | 7.4 ± 2.2 | HPLC | [87] |
| C57BL/6J | f | 4 wks | 8 | c | 50 | 17 | 6.1 | 6.6 | 4.2 | 2.2 | 7.4 ± 2.2 | HPLC | [88] |
| C57BL/6 | m | 8 wks | 12 | c | 50 | 6 | 7.1 | 8.4 | 3.1 | 2.3 | 7.2 ± 0.6 | IA | [89] |
| C57BL/6 | m | 8 wks | 12 | e | 50 | 6 | 7.1 | 1.2 | 3.1 | 2.3 | 13.2 ± 3.2 | IA |  |
| C57BL/6 | m | 60 wks | 14 | c | 50 | 6 | 7.1 | 8.4 | 3.1 | 2.3 | 7.4^#^ | IA |  |
| C57BL/6 | m | 60 wks | 14 | e | 50 | 6 | 7.1 | 1.2 | 3.1 | 2.3 | 11.8^#^ | IA |  |
| C57BL/6J | m | nda | 18 | c | 25 | 7 | 2 | 0 | 3 | 2.5 | 3.2 ± 1.6 | HPLC | [90] |
| C57BL/6J | nda | nda | 16 | c | nda | nda | nda | nda | nda | nda | 2.8^#^ | HPLC | [91] |
| C57BL/6 | m | 6 wks | 4 | c | 25 | 7 | 2 | 0 | 3 | 2.5 | 2.6 ± 0.8 | IA | [12] |
| C57BL/6 | m | 6 wks | 4 | e | 25 | 7 | 2 | 14.8 | 3 | 2.2 | 29.8 ± 3.6 | IA |  |
| C57BL/6 | m | 6 wks | 8 | c | 25 | 7 | 2 | 0 | 3 | 2.5 | 2.5 ± 0.7 | IA |  |
| C57BL/6 | m | 6 wks | 8 | e | 25 | 7 | 2 | 14.8 | 3 | 2.2 | 23.5 ± 1.5 | IA |  |
| C57BL/6 | m | 3 wks | 9 | c | 25 | 6 | 2 | 0 | 2.4 | 1 | 8^#^ | HPLC | [92] |
| C57BL/6 | m | 3 wks | 9 | e1 | 25 | 6 | 2 | 5 | 2.4 | 1 | 14^#^ | HPLC |  |
| C57BL/6 | m | 3 wks | 9 | e2 | 25 | 6 | 2 | 10 | 2.4 | 1 | 17.5^#^ | HPLC |  |
| C57BL/6 | m | 3 wks | 17 | c | 25 | 6 | 2 | 0 | 2.4 | 1 | 11.5^#^ | HPLC |  |
| C57BL/6 | m | 3 wks | 17 | e1 | 25 | 6 | 2 | 5 | 2.4 | 1 | 12.5^#^ | HPLC |  |
| C57BL/6 | m | 3 wks | 17 | e2 | 25 | 6 | 2 | 10 | 2.4 | 1 | 22^#^ | HPLC |  |
| C57BL/6 | m | 3 wks | 26 | c | 25 | 6 | 2 | 0 | 2.4 | 1 | 5^#^ | HPLC |  |
| C57BL/6 | m | 3 wks | 26 | e1 | 25 | 6 | 2 | 5 | 2.4 | 1 | 10^#^ | HPLC |  |
| C57BL/6 | m | 3 wks | 26 | e2 | 25 | 6 | 2 | 10 | 2.4 | 1 | 21^#^ | HPLC |  |
| C57BL/6J | m | 3-4 wks | 15-21 | c | 50 | 7 | 7.5 | 8.2 | 3.5 | 2 | 7^#^ | HPLC | [93] |
| C57BL/6 | m | 6 wks | 8 | c | 25 | 7 | 2 | 0 | 3 | 2.5 | 3.3 ± 0.8 | IA | [16] |
| C57BL/6 | m | 6 wks | 8 | e | 25 | 7 | 2 | 14.8 | 3 | 2.5 | 20.6 ± 2.9 | IA |  |
| C57BL/6 | nda | 3-4 wks | 29-49 | c | 91 | 16.9 | 6.7 | 4 | 3 | 1.7 | 5 ± 0.3 | HPLC | [94] |
| C57BL/6J | f | 12 wks | 6 | c | 30 | 7 | 2 | 3 | 0 | 0 | 10^#^ | IA | [95] |
| C57BL/6J | f | 12 wks | 6 | e | 30 | 7 | 2 | 12 | 0 | 0 | 80^#^ | IA |  |
| C57BL/6 | nda | 3-4 wks | 3-9 | c | 25 | 8.5 | 4 | 4 | 0 | 2 | 2.3 ± 0.4 | HPLC | [96] |
| C57BL/6 | nda | 3-4 wks | 3-9 | e | 25 | 8.5 | 0.2 | 8.2 | 0 | 2 | 7 ± 1.1 | HPLC |  |
| C57BL/6 | f + m | 26 wks | 26 | c | 50 | 6 | 7.1 | 6.7 | 3.1 | 2.3 | 6.9 ± 0.8 | nda | [97] |
| C57BL/6 | f + m | 26 wks | 26 | e | 0 | 0 | 0 | 7.7 | 0 | 2.5 | 68.2 ± 12.1 | nda |  |
| C57BL/6 | m | 5-6 wks | 8 | c | 20 | 6 | 4 | 3 | 10 | 1.3 | 5^#^ | IA | [98] |
| C57BL/6J | nda | 3-4 wks | 12 | c | 25 | 8.5 | 4 | 4 | 0 | 2 | 2.7 ± 0.2 | HPLC | [99] |
| C57BL/6J | nda | 3-4 wks | 12 | e | 25 | 8.5 | 0.2 | 8.2 | 0 | 2 | 7 ± 0.9 | HPLC |  |
| C57BL/6J | m | 6 wks | 15 | c | nda | nda | nda | nda | nda | nda | 3^#^ | AAA | [100] |
| C57BL/6 | m | 6 wks | 4 | c | 12.5 | 7 | 1 | 0 | 0 | 2.5 | 5^#^ | HPLC | [101] |
| C57BL/6 | m | 6 wks | 4 | e | 12.5 | 7 | 1 | 20 | 0 | 2.5 | 61^#^ | HPLC |  |
| C57BL/6 | m | 6 wks | 8 | c | 12.5 | 7 | 1 | 0 | 0 | 2.5 | 10^#^ | HPLC |  |
| C57BL/6 | m | 6 wks | 8 | e | 12.5 | 7 | 1 | 20 | 0 | 2.5 | 48^#^ | HPLC |  |
| C57BL/6 | m | 6 wks | 12 | c | 12.5 | 7 | 1 | 0 | 0 | 2.5 | 10^#^ | HPLC |  |
| C57BL/6 | m | 6 wks | 12 | e | 12.5 | 7 | 1 | 20 | 0 | 2.5 | 50^#^ | HPLC |  |
| C57BL/6 | f | 7 wks | 2 | c | 25 | 7 | 2 | 0 | 3 | 2.5 | 16.7 ± 1.5 | IA | [20] |
| C57BL/6 | f | 7 wks | 2 | e1 | 25 | 7 | 5 | 0 | 3 | 2.5 | 14.8 ± 1.2 | IA |  |
| C57BL/6 | f | 7 wks | 2 | e2 | 25 | 7 | 40 | 0 | 3 | 2.5 | 13.5 ± 1.3 | IA |  |
| C57BL/6J | m | 12-20 wks | 4 | c | 60 | 13 | 2 | 4.4 | 3.9 | 1.9 | 8.0 ± 0.4 | IA | [102] |
| C57BL/6J | m | 12-20 wks | 20 | c | 60 | 13 | 2 | 4.4 | 3.9 | 1.9 | 8.6 ± 0.8 | IA |  |
| C57BL/6J | f | 6-8 wks | 6 | c | 25 | 7 | 1.2 | 0 | 1.8 | 2.5 | 21 ± 3.7 | HPLC | [103] |
| C57BL/6J | f | 6-8 wks | 6 | e1 | 25 | 7 | 0.1 | 0 | 1.8 | 2.5 | 24.5 ± 2.5 | HPLC |  |
| C57BL/6J | f | 6-8 wks | 6 | e2 | 25 | 7 | 0.1 | 0 | 1.8 | 2.5 | 47.4 ± 7.8 | HPLC |  |
| C57BL/6J | m | 17 wks | 10 | c | 5 | 7 | 2 | nda | nda | nda | 11.4 ± 0.8 | HPLC | [104] |
| C57BL/6J | m | 17 wks | 10 | e1 | 5 | 7 | 0 | nda | nda | nda | 16.2 ± 1.6 | HPLC |  |
| C57BL/6J | m | 17 wks | 10 | e2 | 5 | 7 | 0 | nda | nda | nda | 13.3 ± 1.5 | HPLC |  |
| C57BL/6J | m | 17 wks | 10 | e3 | 5 | 0.5 | 0 | nda | nda | nda | 15.1 ± 1.6 | HPLC |  |
| C57BL/6J | m | 17 wks | 10 | e4 | 0 | 7 | 0 | nda | nda | nda | 13.8 ± 0.8 | HPLC |  |
| C57BL/6J | m | 17 wks | 10 | e5 | 0 | 0.5 | 0 | nda | nda | nda | 16 ± 1.5 | HPLC |  |
| C57BL/6 | f | 3-4 wks | 3-4 | c | 19.2 | 18.9 | 3 | 3.7 | 3.5 | 1.4 | 6^#^ | HPLC | [105] |
| C57BL/6 | m | 3-4 wks | 3-4 | c | 19.2 | 18.9 | 3 | 3.7 | 3.5 | 1.4 | 6^#^ | HPLC |  |
| C57BL/6J | f | 3-4 wks | 2-5 | c | 90 | 17 | 7 | 4 | 3 | 2.2 | 5.5 | GC or HPLC | [106] |
| C57BL/6N | m | 4 wks | 12 | c | 30 | 18 | 16 | 6.5 | 2.7 | 1 | 10.1 ± 1 | HPLC | [107] |
| C57BL/6N | m | 4 wks | 12 | e | 30 | 18 | 0.1 | 6.5 | 2.7 | 1 | 13.5 ± 2.2 | HPLC |  |
| C57BL/6 | f | 10-12 wks | 39-47 | c | 51 | 10 | 1.9 | 6.2 | 3.6 | 2 | 5^#^ | nda | [108] |
| C57BL/6 | f_p_ | 3-4 wks | 6 | c | 25 | 7 | 2 | 3.3 | 3.5 | 2.5 | 14.5^#^ | IA | [109] |
| C57BL/6 | m | 3 wks | 0 | c | 25 | 7 | 2 | 3.3 | 3.5 | 2.5 | 6^#^ | IA |  |
| C57BL/6 | f | 3.5 wks | 15.5 | c | nda | nda | 2 | nda | nda | nda | 5.0 ± 1.2 | HPLC | [110] |
| C57BL/6 | f | 3.5 wks | 15.5 | e | nda | nda | 0.3 | nda | nda | nda | 10.2 ± 1.5 | HPLC |  |
| C57BL/6JRj | m | 9 wks | 6 | c | 50 | 7 | 8 | 0 | 0 | 2 | 4.9 ± 0.4 | HPLC | [111] |
| C57BL/6JRj | m | 9 wks | 6 | e | 50 | 7 | 0 | 0 | 0 | 2 | 11.4 ± 0.9 | HPLC |  |
| FVB | f | 3 wks | 3 | c | 50 | 7 | 2 | 8.2 | 3.5 | 2 | 20^#^ | HPLC | [112] |
| FVB | f | 3 wks | 3 | e1 | 50 | 7 | 0 | 8.2 | 3.5 | 2 | 290^#^ | HPLC |  |
| FVB | f | 3 wks | 3 | e2 | 50 | 7 | 5 | 8.2 | 3.5 | 2 | 20^#^ | HPLC |  |
| FVB | f | 3 wks | 3 | e3 | 50 | 7 | 20 | 8.2 | 3.5 | 2 | 20^#^ | HPLC |  |
| Swiss | f | adult | 3 | c | 25 | 7 | 2 | 0 | 1.8 | 2.5 | 9.2 | HPLC | [14] |
| Swiss | f | adult | 3 | e | 2.4 | nda | 0.3 | nda | nda | 0 | 13.6 | HPLC |  |
| Swiss | f | 3 wks | 1 | c | 25 | 7 | 2 | 0 | 1.8 | 2.5 | 9.7 ± 0.6 | HPLC |  |
| Swiss | f | 3 wks | 10 | c | 25 | 7 | 2 | 0 | 1.8 | 2.5 | 7.1 ± 0.7 | HPLC |  |
| Swiss | f | 3 wks | 27 | c | 25 | 7 | 2 | 0 | 1.8 | 2.5 | 8.1 ± 0.8 | HPLC |  |
| Swiss | m | 3 wks | 1 | c | 25 | 7 | 2 | 0 | 1.8 | 2.5 | 8.7 ± 0.9 | HPLC |  |
| Swiss | m | 3 wks | 10 | c | 25 | 7 | 2 | 0 | 1.8 | 2.5 | 5.2 ± 0.6 | HPLC |  |
| Swiss | m | 3 wks | 27 | c | 25 | 7 | 2 | 0 | 1.8 | 2.5 | 3.0 ± 0.4 | HPLC |  |
| Swiss Webster | f | weanling | 7 | c | 50 | 7 | 11.3 | 8.2 | 3.5 | 2 | 9 ± 1 | HPLC | [113] |
| Swiss Webster | f | weanling | 7 | e | 50 | 7 | 0 | 8.2 | 3.5 | 2 | 125 ± 36 | HPLC |  |
| 129/Sv | f + m | 3 wks | 9-13 | c | 25 | 7 | 2 | 0 | 1.8 | 2.5 | 2 ± 0.6 | IA | [10] |
| 129/Sv | f + m | 3 wks | 9-13 | e | 1.8 | 0.9 | 0.1 | 0 | 1.8 | 2.5 | 90.7 ± 34.1 | IA |  |
| 129/Sv | f + m | 3 wks | 6 | c | 25 | 7 | 2 | 0 | 1.8 | 2.5 | 0.05^#^ | IA | [9] |
| 129/Sv | f + m | 3 wks | 6 | e | 1.8 | 0.2 | 0.1 | 0 | 1.8 | 2.5 | 280^#^ | IA |  |
| 129/Sv | f + m | 3 wks | 9 | c | 25 | 7 | 2 | 0 | 1.8 | 2.5 | 0.1^#^ | IA |  |
| 129/Sv | f + m | 3 wks | 9 | e | 1.8 | 0.2 | 0.1 | 0 | 1.8 | 2.5 | 320^#^ | IA |  |
| 129/Sv | nda | 3-4 wks | 8.5 | c | 25 | 7 | 2 | 0 | 1.8 | 2.5 | 5 ± 0.2 | IA | [114] |
| 129/Sv | nda | 3-4 wks | 8.5 | e | 1.8 | 0.9 | 0.1 | 0 | 1.8 | 2.5 | 36.8 ± 5.2 | IA |  |
| 129/Sv | f + m | 3 wks | 9 | c | 25 | 7 | 2 | 0 | 1.8 | 2.5 | 2.8 ± 0.3 | HPLC | [13] |
| 129/Sv | f + m | 3 wks | 9 | e | 1.8 | 0.2 | 0.1 | 0 | 1.8 | 2.5 | 15.1 ± 5.6 | HPLC |  |
| **Studies including different mouse strains** | | | |  |  |  |  |  |  |  |  |  |  |
| A/J | f | 3-4 wks | 3-4 | e1 | 19.8 | 17 | 6 | 4.3 | 4.2 | 2.2 | 3.9 ± 0.2 | HPLC | [115] |
| A/J | f | 3-4 wks | 3-4 | e2 | 81.6 | 9 | 1.8 | 3.5 | 3 | 1.9 | 3.6 ± 0.2 | HPLC |  |
| A/J | m | 3-4 wks | 3-4 | e1 | 19.8 | 17 | 6 | 4.3 | 4.2 | 2.2 | 2.4 ± 0.1 | HPLC |  |
| A/J | m | 3-4 wks | 3-4 | e2 | 81.6 | 9 | 1.8 | 3.5 | 3 | 1.9 | 2.5 ± 0.1 | HPLC |  |
| C57BL/6J | f | 3-4 wks | 3-4 | e1 | 19.8 | 17 | 6 | 4.3 | 4.2 | 2.2 | 4.4 ± 0.3 | HPLC |  |
| C57BL/6J | f | 3-4 wks | 3-4 | e2 | 81.6 | 9 | 1.8 | 3.5 | 3 | 1.9 | 5.9 ± 0.4 | HPLC |  |
| C57BL/6J | m | 3-4 wks | 3-4 | e1 | 19.8 | 17 | 6 | 4.3 | 4.2 | 2.2 | 2.8 ± 0.2 | HPLC |  |
| C57BL/6J | m | 3-4 wks | 3-4 | e2 | 81.6 | 9 | 1.8 | 3.5 | 3 | 1.9 | 4 ± 0.2 | HPLC |  |
| EL/SuZ | f | 3-4 wks | 3 | e1 | 19.8 | 17 | 6 | 4.3 | 4.2 | 2.2 | 3.2 ± 0.4 | HPLC |  |
| NOD/LtJ | f | 3-4 wks | 3 | e1 | 19.8 | 17 | 6 | 4.3 | 4.2 | 2.2 | 3.3 ± 0.1 | HPLC |  |
| DDY/JeL | f | 3-4 wks | 3 | e1 | 19.8 | 17 | 6 | 4.3 | 4.2 | 2.2 | 3.5 ± 0.2 | HPLC |  |
| SWR/J | f | 3-4 wks | 3 | e1 | 19.8 | 17 | 6 | 4.3 | 4.2 | 2.2 | 3.8 ± 0.1 | HPLC |  |
| NOR/LtJ | f | 3-4 wks | 3 | e1 | 19.8 | 17 | 6 | 4.3 | 4.2 | 2.2 | 4.2 ± 0.3 | HPLC |  |
| CBA/CaJ | f | 3-4 wks | 3 | e1 | 19.8 | 17 | 6 | 4.3 | 4.2 | 2.2 | 5.6 ± 0.1 | HPLC |  |
| SWXL-4 | f | 3-4 wks | 3 | e1 | 19.8 | 17 | 6 | 4.3 | 4.2 | 2.2 | 5.6 ± 0.3 | HPLC |  |
| BALB/cByJ | f | 3-4 wks | 3 | e1 | 19.8 | 17 | 6 | 4.3 | 4.2 | 2.2 | 6 ± 0.2 | HPLC |  |
| CBA/J | f | 3-4 wks | 3 | e1 | 19.8 | 17 | 6 | 4.3 | 4.2 | 2.2 | 5.9 ± 0.1 | HPLC |  |
| DBA/2J | f | 3-4 wks | 3 | e1 | 19.8 | 17 | 6 | 4.3 | 4.2 | 2.2 | 6.3 ± 0.1 | HPLC |  |
| C3H/HeJ | f | 3-4 wks | 3 | e1 | 19.8 | 17 | 6 | 4.3 | 4.2 | 2.2 | 6.6 ± 0.4 | HPLC |  |
| ABP/Le | f | 3-4 wks | 3 | e1 | 19.8 | 17 | 6 | 4.3 | 4.2 | 2.2 | 7 ± 0.6 | HPLC |  |
| C57L | m | 6-8 wks | 0 | e | 22 | 6 | 5.9 | 4.3 | 3.2 | 2.3 | 4.3 ± 0.2 | HPLC | [116] |
| C57L | m | 6-8 wks | 2 | e | 22 | 6 | 5.9 | 4.3 | 3.2 | 2.3 | 6.5^#^ | HPLC |  |
| C57L | m | 6-8 wks | 4 | e | 22 | 6 | 5.9 | 4.3 | 3.2 | 2.3 | 8.7 ± 0.7 | HPLC |  |
| C57L | m | 6-8 wks | 7 | e | 22 | 6 | 5.9 | 4.3 | 3.2 | 2.3 | 5.9^#^ | HPLC |  |
| C57L | m | 6-8 wks | 10 | e | 22 | 6 | 5.9 | 4.3 | 3.2 | 2.3 | 6.2^#^ | HPLC |  |
| C57L | m | 6-8 wks | 17 | e | 22 | 6 | 5.9 | 4.3 | 3.2 | 2.3 | 6.1^#^ | HPLC |  |
| C57L | m | 6-8 wks | 22 | e | 22 | 6 | 5.9 | 4.3 | 3.2 | 2.3 | 7^#^ | HPLC |  |
| C57L | m | 6-8 wks | 36 | e | 22 | 6 | 5.9 | 4.3 | 3.2 | 2.3 | 6^#^ | HPLC |  |
| C57L | m | 6-8 wks | 56 | e | 22 | 6 | 5.9 | 4.3 | 3.2 | 2.3 | 6^#^ | HPLC |  |
| C57BL/6 | m | 6-8 wks | 0 | e | 22 | 6 | 5.9 | 4.3 | 3.2 | 2.3 | 4.2^#^ | HPLC |  |
| C57BL/6 | m | 6-8 wks | 6 | e | 22 | 6 | 5.9 | 4.3 | 3.2 | 2.3 | 7.8^#^ | HPLC |  |
| C57BL/6 | m | 6-8 wks | 9 | e | 22 | 6 | 5.9 | 4.3 | 3.2 | 2.3 | 7.4^#^ | HPLC |  |
| C57BL/6 | m | 6-8 wks | 16 | e | 22 | 6 | 5.9 | 4.3 | 3.2 | 2.3 | 7.5^#^ | HPLC |  |
| C57BL/6 | m | 6-8 wks | 21 | e | 22 | 6 | 5.9 | 4.3 | 3.2 | 2.3 | 3.5^#^ | HPLC |  |
| SWR | m | 6-8 wks | 0 | e | 22 | 6 | 5.9 | 4.3 | 3.2 | 2.3 | 5.2^#^ | HPLC |  |
| SWR | m | 6-8 wks | 6 | e | 22 | 6 | 5.9 | 4.3 | 3.2 | 2.3 | 7.5^#^ | HPLC |  |
| SWR | m | 6-8 wks | 9 | e | 22 | 6 | 5.9 | 4.3 | 3.2 | 2.3 | 9^#^ | HPLC |  |
| SWR | m | 6-8 wks | 16 | e | 22 | 6 | 5.9 | 4.3 | 3.2 | 2.3 | 4.8^#^ | HPLC |  |
| SWR | m | 6-8 wks | 21 | e | 22 | 6 | 5.9 | 4.3 | 3.2 | 2.3 | 6^#^ | HPLC |  |
| AKR | m | 6-8 wks | 0 | e | 22 | 6 | 5.9 | 4.3 | 3.2 | 2.3 | 4.5^#^ | HPLC |  |
| AKR | m | 6-8 wks | 2 | e | 22 | 6 | 5.9 | 4.3 | 3.2 | 2.3 | 4^#^ | HPLC |  |
| AKR | m | 6-8 wks | 4 | e | 22 | 6 | 5.9 | 4.3 | 3.2 | 2.3 | 5.7^#^ | HPLC |  |
| AKR | m | 6-8 wks | 7 | e | 22 | 6 | 5.9 | 4.3 | 3.2 | 2.3 | 5.8^#^ | HPLC |  |
| AKR | m | 6-8 wks | 10 | e | 22 | 6 | 5.9 | 4.3 | 3.2 | 2.3 | 5.2^#^ | HPLC |  |
| AKR | m | 6-8 wks | 17 | e | 22 | 6 | 5.9 | 4.3 | 3.2 | 2.3 | 5^#^ | HPLC |  |
| AKR | m | 6-8 wks | 22 | e | 22 | 6 | 5.9 | 4.3 | 3.2 | 2.3 | 4.1^#^ | HPLC |  |
| AKR | m | 6-8 wks | 36 | e | 22 | 6 | 5.9 | 4.3 | 3.2 | 2.3 | 4^#^ | HPLC |  |
| AKR | m | 6-8 wks | 56 | e | 22 | 6 | 5.9 | 4.3 | 3.2 | 2.3 | 5.3^#^ | HPLC |  |
| A/J | m | 6 wks | 12 | e | 10 | 7 | 0 | 1.7 | 3.7 | 0 | 25^#^ | HPLC | [117] |
| C57BL/6J | m | 6 wks | 12 | e | 10 | 7 | 0 | 1.7 | 3.7 | 0 | 24^#^ | HPLC |  |
| C3H/HeJ | m | 6 wks | 12 | e | 10 | 7 | 0 | 1.7 | 3.7 | 0 | 26^#^ | HPLC |  |
| CAST/EiJ | m | 6 wks | 12 | e | 10 | 7 | 0 | 1.7 | 3.7 | 0 | 15^#^ | HPLC |  |
| 129S1/SvImJ |  | 6 wks | 12 | e | 10 | 7 | 0 | 1.7 | 3.7 | 0 | 29^#^ | HPLC |  |
| PWK/PhJ | m | 6 wks | 12 | e | 10 | 7 | 0 | 1.7 | 3.7 | 0 | 34^#^ | HPLC |  |
| WSB/EiJ | m | 6 wks | 12 | e | 10 | 7 | 0 | 1.7 | 3.7 | 0 | 47^#^ | HPLC |  |
| BALB/c | f + m | weanling | 52 | c | 25 | 7 | 2 | 3.3 | 3.5 | 2.5 | 8.6 ± 1.2 | HPLC | [118] |
| BALB/c | f + m | weanling | 52 | e | 25 | 7 | 0.3 | 3.3 | 3.5 | 2.5 | 18.9 ± 3.6 | HPLC |  |
| C57BL/6 | f + m | weanling | 52 | c | 25 | 7 | 2 | 3.3 | 3.5 | 2.5 | 5 ± 0.1 | HPLC |  |
| C57BL/6 | f + m | weanling | 52 | e | 25 | 7 | 0.3 | 3.3 | 3.5 | 2.5 | 9.9 ± 1.2 | HPLC |  |
| SAMP8 | m | 13 wks | 4 | c | 25 | 7 | 2 | 0 | 1.8 | 2.5 | 4^#^ | IA | [18] |
| SAMP8 | m | 17 wks | 26 | c | 25 | 7 | 2 | 0 | 1.8 | 2.5 | 6.5^#^ | IA |  |
| SAMP8 | m | 17 wks | 26 | e1 | 25 | 7 | 0 | 0 | 1.8 | 2.5 | 17^#^ | IA |  |
| SAMP8 | m | 17 wks | 26 | e2 | 25 | 7 | 2.5 | 0 | 1.8 | 2.5 | 5.8^#^ | IA |  |
| SAMP8 | m | 17 wks | 26 | e3 | 25 | 7 | 3 | 0 | 1.8 | 2.5 | 5.6^#^ | IA |  |
| SAMR1 | m | 17 wks | 26 | c | 25 | 7 | 2 | 0 | 1.8 | 2.5 | 6.5^#^ | IA |  |
| **Mouse strain not mentionend** | | |  |  |  |  |  |  |  |  |  |  |  |
| nda | f | 12-20 wks | nda | c | 50 | 9.3 | 3.0 | 7 | 2.8 | 2.5 | 6.5^#^ | HPLC | [119] |
| nda | m | 12-20 wks | nda | c | 50 | 9.3 | 3.0 | 7 | 2.8 | 2.5 | 7.7^#^ | HPLC |  |
| nda | f + m | 3-4 wks | 9-36 | c | 90 | 17 | 7 | 4 | 3 | 2.2 | 7.4 ± 2.9 | HPLC | [120] |
| nda | f | 31 wks | nda | c | nda | nda | nda | nda | nda | nda | 5.8 ± 0.6 | IA | [121] |
| nda | m | 52-69 wks | nda | c | nda | nda | nda | nda | nda | nda | 5.9 ± 0.6 | IA |  |
| nda | nda | nda | nda | c | nda | nda | nda | nda | nda | nda | 3.9 ± 0.9 | IA | [122] |
| nda | nda | nda | nda | c | nda | nda | nda | nda | nda | nda | 3.9 ± 0.9 | IA | [123] |
| nda | nda | nda | nda | c | nda | nda | nda | nda | nda | nda | 3.9 ± 0.9 | IA | [124] |
| nda | nda | nda | nda | c | nda | nda | nda | nda | nda | nda | 4.4^#^ | HPLC | [125] |
| nda | nda | nda | nda | c | nda | nda | nda | nda | nda | nda | 4.1 ± 0.3 | HPLC | [126] |
| nda | f | 13-17 wks | 4 | c | 51 | 5.4 | 0.9 | 3.3 | 2.7 | 1.8 | 2.9 ± 2 | HPLC | [127] |
| nda | f_p_ | 13-17 wks | 4 | c | 51 | 5.4 | 0.9 | 3.3 | 2.7 | 1.8 | 6.1 ± 3 | HPLC |  |
| nda | f | 6-8 wks | nda | c | nda | nda | 4 | nda | nda | nda | 5.1 + 0.9 | HPLC | [128] |
| nda |  | nda | nda | e1 | nda | nda | 0 | nda | nda | nda | 26.5 + 16.5 | HPLC |  |
| nda |  | nda | nda | e2 | nda | nda | 10 | nda | nda | nda | 9.3 + 4.0 | HPLC |  |

Diet composition (concentrations of vitamin B_12_ (µg/kg), vitamin B_6_ (mg/kg), folate (mg/kg), methionine (g/kg), cystine (g/kg) and choline (g/kg) in control (c) and experimental (e) diets) and mouse data (strain, sex (f_female, f_p__pregnant female, m_male), age or weight at baseline, duration of dietary intervention, homocysteine levels (^#^_read from diagrams), and detection method (AAA_amino acid analyzer, CHEM_chemiluminescence, GC_gas chromatography, HPLC_high performance liquid chromatography, IA_immunoassay)); nda_no data available.
